# Developing a semantically rich ontology for the biobank-administration domain

**DOI:** 10.1186/2041-1480-4-23

**Published:** 2013-10-08

**Authors:** Mathias Brochhausen, Martin N Fransson, Nitin V Kanaskar, Mikael Eriksson, Roxana Merino-Martinez, Roger A Hall, Loreana Norlin, Sanela Kjellqvist, Maria Hortlund, Umit Topaloglu, William R Hogan, Jan-Eric Litton

**Affiliations:** 1 Division of Biomedical Informatics, University of Arkansas for Medical Sciences, Little Rock, AR, USA; 2 Department of Medical Epidemiology and Biostatistics, Karolinska Institutet, Stockholm, Sweden; 3 Department of IT Research, University of Arkansas for Medical Sciences, Little Rock, AR, USA

## Abstract

**Background:**

Biobanks are a critical resource for translational science. Recently, semantic web technologies such as ontologies have been found useful in retrieving research data from biobanks. However, recent research has also shown that there is a lack of data about the administrative aspects of biobanks. These data would be helpful to answer research-relevant questions such as what is the scope of specimens collected in a biobank, what is the curation status of the specimens, and what is the contact information for curators of biobanks. Our use cases include giving researchers the ability to retrieve key administrative data (e.g. contact information, contact's affiliation, etc.) about the biobanks where specific specimens of interest are stored. Thus, our goal is to provide an ontology that represents the administrative entities in biobanking and their relations. We base our ontology development on a set of 53 data attributes called MIABIS, which were in part the result of semantic integration efforts of the European Biobanking and Biomolecular Resources Research Infrastructure (BBMRI). The previous work on MIABIS provided the domain analysis for our ontology. We report on a test of our ontology against competency questions that we derived from the initial BBMRI use cases. Future work includes additional ontology development to answer additional competency questions from these use cases.

**Results:**

We created an open-source ontology of biobank administration called *Ontologized MIABIS* (OMIABIS) coded in OWL 2.0 and developed according to the principles of the OBO Foundry. It re-uses pre-existing ontologies when possible in cooperation with developers of other ontologies in related domains, such as the Ontology of Biomedical Investigation. OMIABIS provides a formalized representation of biobanks and their administration. Using the ontology and a set of Description Logic queries derived from the competency questions that we identified, we were able to retrieve test data with perfect accuracy. In addition, we began development of a mapping from the ontology to pre-existing biobank data structures commonly used in the U.S.

**Conclusions:**

In conclusion, we created OMIABIS, an ontology of biobank administration. We found that basing its development on pre-existing resources to meet the BBMRI use cases resulted in a biobanking ontology that is re-useable in environments other than BBMRI. Our ontology retrieved all true positives and no false positives when queried according to the competency questions we derived from the BBMRI use cases. Mapping OMIABIS to a data structure used for biospecimen collections in a medical center in Little Rock, AR showed adequate coverage of our ontology.

## Introduction

Biobanks are a critical resource in translational science, such as translational oncology, as they provide specimens essential to the identification of novel biomarkers for specific therapies [1]. Recent research has provided compelling examples of using semantic web technologies, such as ontologies, to retrieve research-relevant data from biobanks [2,3]. However, [4] point out that little attention is paid to collecting data about the different ways in which biobanks are organized. This lack is apparent in both of the ontologies considered by the authors of [2,3]: Neither the Ontology of Biomedical Investigation (OBI)^a^, nor the Translational Medicine Ontology (TMO)^b^ represent biobanks, biobank organizations, or related entities. This situation makes it impossible to query biobanks with respect to organizational structures, ownership of biobanks and specimens, and the curation status of specimens. Thus, our goal was to provide an ontology that represents the administrative aspects of the biobanking domain to enable querying biobank data from both the specimen or population perspective and the administrative perspective. Our ontology is called *Ontologized MIABIS* (OMIABIS) and is named after the *Minimum Information About BIobank data Sharing* (MIABIS) [5]. The latter provided the starting point for our ontology development. We recently released the initial version of OMIABIS coded in Web Ontology Language 2.0. It can be downloaded from http://purl.obolibrary.org/obo/omiabis.owl. The ontology is open source and we invite the community to develop it further with us.

In the background section we introduce MIABIS and its use cases. In the methods section we describe our approach to ontology development including the re-use of existing ontologies. In addition, we introduce our approach to testing the ability of the ontology to answer competency questions derived from our use cases. In the results section, we show the basic features of our ontology and present the results of our evaluation of its adequacy. Finally, we discuss future work and potential uses of the ontology, as well as its connections to ongoing efforts in biomedical ontology.

## Background

### Introducing BBMRI

For an initial domain analysis we relied on the work on data integration done by the European Biobanking and Biomolecular Resources Research Infrastructure (BBMRI). During the so-called preparatory phase of BBMRI, between 2008–2011, the initiative comprised 54 different partners across Europe and more than 225 associated organizations representing over 30 countries. One of the aims of the BBMRI is to provide the necessary formats to compare biobank information at different levels of detail [6]. Work on data integration within BBMRI used at least two approaches; a survey of the samples and data of European biobanks using questionnaires—resulting in the Catalogue of European Biobanks [7], and the development of a common information model for a hub-and-spokes structure for national or regional biobank nodes [8]. Because biobank data is often related to personal health data, management and sharing must follow legal jurisdiction, according to Directive 95/46/EC in the European context. In combination with several other integration issues identified in [9], the establishment of an information model for sharing biobank data on an international level will require future effort. In the meantime, and to meet the demand of the biobank community to understand what data should be stored in relation to biological samples, a minimum list of data attributes was drafted as one of the last activities in the preparatory phase of BBMRI. One of the activities in the Swedish BBMRI, i.e., BBMRI.se, has been to continue the development of the minimum information list from the European BBMRI. The updated version is called MIABIS – Minimum Information About BIobank data Sharing – and consists of fifty-two attributes considered important for establishing a system of data discovery for biobanks and sample collections. To avoid legal issues related to individual subjects, cases or samples are not considered at present [5]. The attributes employ existing standards, e.g., the Sample PREanalytical Code (SPREC) [10], ICD Codes^c^, and definitions developed by the Public Population Project in Genomics (P^3^G)^d^ and the International Society for Biological and Environmental Repositories (ISBER)^e^.

### Use cases for MIABIS & OMIABIS

MIABIS was developed in the context of several use cases described by invited researchers as part of the BBMRI project. Our two example use cases stem from the development of MIABIS:

a) Search for tissue samples from donors diagnosed with nemaline myopathy. Determine the age group. What are the sample storage conditions? Contact the biobank for detailed information about the biopsy samples and whether myoblast cell cultures have been grown from these samples.

b) Search for sample collections having at least 10 cases with tissue from the thoracic aorta as well as blood, serum, or plasma from the same donor. Also check if clinical data has been registered for the donors such as physical measurements. Contact the person responsible for the sample collection to obtain detailed information on the specific kind of thoracic aorta biopsies of interest. Also assure that the biopsies were performed +/- one week in relation to the blood sampling.

Use case b) would require inclusion of individual-level data. As mentioned above, the attributes for representing data about individual donors and specimens were dropped during MIABIS development due to regulatory issues.

Already, MIABIS is being used in a structured Scandinavian survey to gather information about sample collections stored in biobanks in a searchable database (http://www.bbmriregister.se). Increasing the total searchable information could include uploading new data directly to the existing system, and/or developing external databases that structure the information according to MIABIS. In the latter case, an ontologized version of MIABIS will be used to perform a federated search across the multiple databases. This search capability will minimize the effort a researcher must expend to search for sample collections of interest, by avoiding the need to query several separate databases one by one. Hence, the University of Arkansas for Medical Sciences and Karolinska Institutet, representing BBMRI.se, decided to initiate a biobank ontology development project as a joint effort.

## Methods

Our aim is to provide a semantically rich representation of biobank administration to facilitate the sharing of biobank data. We based our development on an analysis of the minimum requirements for sharing biobank data done within the BBMRI as captured by MIABIS. Hence, we named our ontology OMIABIS, standing for *Ontologized MIABIS*. To make the ontology easily accessible and implementable, we chose Web Ontology Language (OWL) 2 [11] for implementation. To facilitate re-use and harmonization across ontologies, we used Basic Formal Ontology (BFO)^f^ as the upper ontology [12,13]. In addition, the entire ontology development followed the principles of ontology development as set forth by the OBO Foundry [14]^g^.

Re-use of preexisting ontologies is key among the OBO Foundry principles. In creating OMIABIS we imported the Proper Name Ontology (PNO)^h^ in its entirety. PNO is based on the Information Artifact Ontology (IAO)^i^. It is a formal representation of proper names based on Devitt's theory of designation [15]. Thus, OMIABIS is an extension of IAO. In addition, multiple entities from other ontologies, namely the Ontology of Biomedical Investigations (OBI)^j^ and the Ontology of Medically Relevant Social Entities (OMRSE)^k^ are imported using a tool based on the MIREOT methodology [16], which was developed in a joint endeavor between the University of Arkansas for Medical Sciences and the University of Arkansas at Little Rock [17].

We chose to re-use the ontologies mentioned above based on the fact that they are members of the OBO Foundry and, thus, are built according to the same basic principles and extend the same upper ontology (BFO). Our aim is to create ontological representations that facilitate the integration of biobank administrative data with biomedical research data. The latter often is annotated with terms from Gene Ontology (GO) or OBI. Thus, choosing ontologies from the very same orthogonal ontology library (OBO Foundry) of which the latter are members appears to be the best strategy to accomplish this integration.

All directly imported ontologies (BFO, PNO, IAO) will update automatically. MIREOT, so far, does not have a strategy for automated updates. However, the developers of the MIREOT plugin plan to include this functionality in a future release.

In addition to these ontologies, the development of OMIABIS was informed by other pre-existing ontologies in the biobanking domain mentioned in the Discussion section of this paper.

Because existing ontologies already represent specimens, clinical studies and populations, OMIABIS represents the domain of biobank administration. Together with terms from these specimen-focused ontologies, OMIABIS needs to allow the level of semantic integration required by the use cases described above.

OMIABIS was developed using Protégé 4.1.0, Build 239^l^. The MIREOT Plugin is Version 1.0.1. The consistency of our ontology was verified using the HermiT 1.3.6 reasoner^m^.

To test the adequacy of our ontology for the BBMRI use cases (s. Background section) we derived a set of competency questions from them. Because the focus of the ontology is the administrative aspects of biobanks, the use cases entail some competency questions that fall outside the scope of our ontology at this point (namely all questions related to the different donor subpopulations).

The competency questions we address and evaluate here are:

•Which biobanks hold frozen specimens?

•Which biobanks hold blood, plasma and serum?

•Which blood plasma specimens are owned by one specific biobank organization?

•Which departments of a specific university have members that are serving as biobank contacts?

•What are the e-mail addresses of all biobank contact persons at one specific biobank organization?

These competency questions were approved by the domain experts from Karolinska Institute.

To perform DL queries that test the adequacy of the ontology to retrieve data that answer the competency questions, we populated an OWL file (that imports OMIABIS) with instances or individuals from a made-up biobank example. In OWL it is possible to represent the individual members of classes. OMIABIS per se does not represent any individuals, but it imports 326 individuals from GEO that represent nations and their administrative subdivisions (to enable capture of the mailing addresses of biobank contacts). We included both true positives and false positives to the instance-level OWL file, to ensure that queries did not retrieve incorrect information. This file is called CompetencyTest.owl, and can be downloaded from: http://omiabis-dev.googlecode.com/svn/branches/CompetencyTest.owl. In addition, we submitted the file to this journal as Additional file 1.

The actual queries we ran together with the results can be found in Table 1.

**Table 1:** DL Queries executed on the Competency Test OWL file and results

**Competency question**	**DL Query**	**Recall**	**Precision**	**Ratio**	**Reasoning time (in ms)**
Which biobanks hold frozen specimens?	biobank and has_part some 'frozen specimen'	100%	100%	6/6	76.9
Which biobanks hold blood, plasma and serum?	biobank and has_part some 'blood plasma specimen' and has_part some 'blood serum specimen' and has_part some 'blood specimen'	100%	100%	5/5	53.2
Which blood plasma specimens are owned by one specific biobank organization?	'blood plasma specimen' and part_of some (biobank and 'is owned by' some {'Unseen University'})	100%	100%	6/6	45.4
Which departments of a specific university have members that are serving as biobank contacts?	department and 'has organization member' some (bearer_of some 'biobank contact role')	100%	100%	6/6	30.2
What are the e-mail addresses of all biobank contact persons at one specific biobank organization?	'email address' and 'is contact information about' some (bearer_of some 'biobank contact role' and 'is member of organization' some {'Unseen University'})	100%	100%	6/6	55.2

Each query is encoded in a separate test method in Scala using the Java OWL-API. Each method reloads the ontology, builds the necessary axiom from API calls, adds the axiom, executes the query, and removes the axiom from the ontology. Each trial was conducted by running each method 100 times and reporting the average running time in milliseconds. Ten trials were conducted.

## Results

### Implementation of OMIABIS

The latest release of OMIABIS in OWL can be downloaded from the permanent URL http://purl.obolibrary.org/obo/omiabis.owl. In our research we focused on representing the MIABIS data attributes focused on biobanks and studies/sample collections, which comprises all classes and object properties closely related to administrative aspects.

The central class of any biobank ontology ought to be the class of biobanks or biorepositories. MIABIS differentiates biobanks from the organizations that own them. Accordingly, OMIABIS defines "biobank" as follows: "A biobank is a collection of samples of biological substances (e.g. tissue, blood, DNA) which are linked to data about the samples and their donors. They have a dual nature as collections of samples and data." The definition is derived from the definition for human biobank in [18]. The latter does not define "biobank” in general, but we generalized their definition to be applicable to any kind of biobank. The class is formally restricted to be the equivalent of^n^:


Notably, the biobank as such is neither an organization nor a facility, but the aggregate of the specimens and the data regarding these specimens. OMIABIS also represents "biobank organization". Its textual definition is: "A biobank organization is an organization bearing legal personality that owns or administrates a biobank". "Biobank organization" is equivalent to:


Referring to the class "legal person role" from OMRSE is necessary due to the fact that the definition of organization in OBI does not refer to legal personality^p^. Any group of human beings that has some organizational rules fulfills the textual definition according to OBI. However, for our use case legal personality is crucial, since within the BBMRI framework we are concerned with management of certain rights and obligations, which are held by legal persons. The formal description of biobank organization uses two object properties which have been specifically created for OMIABIS:

1. "*owns*"

Elucidation: This is a primitive relation. This relation is the foundation to the owner’s right to have the owned entity at his/her full disposal.


Domain: **Homo sapiens**

OR **organization**

OR **collection of humans**

OR **aggregate of organizations**

Range: **information content entity**

OR **material_entity**

Characteristics: asymmetric

The elucidation for this primitive relation is based on Reinach's legal ontology [19]. For further material on the ontology of claims and obligations see [20].

2. "*administrates*"

Definition: "a administrates b if c owns b and some rights and obligations grounded in the owning relation regarding b are transferred ^[q]^

The 'transfers' object property is represented in Document Acts Ontology (d-acts): http://purl.obolibrary.org/iao/d-acts.owl

from c to a."

Domain: **Homo sapiens**

OR **organization**

OR **collection of humans**

OR **aggregate of organizations**

Range: **information content entity**

OR **material_entity**

Characteristics: asymmetric

**Figure 1:**
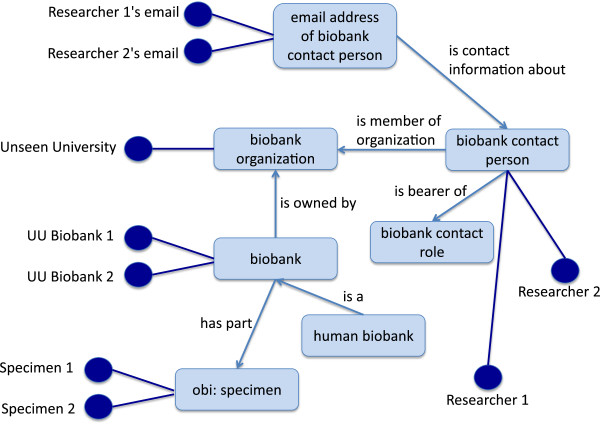
**Illustration of the central OMIABIS classes.** The figure shows the central classes of OMIABIS and the object properties connecting them. Light blue rectangles are classes; light blue arrows are object properties. Dark blue circles and edges represent instances that can be retrieved using OMIABIS.

OMIABIS includes a total of 249 classes and 64 object properties. Of the 249 classes 34 classes are restricted by an equivalent class axiom. 35 classes and object properties were newly created for the initial version of OMIABIS. A textual definition is given for all newly created classes and object properties. Figure 1 shows a semantic network for the central classes of OMIABIS and how they are used in retrieving data matching the competency questions.

The OMIABIS labels tend to be very long, since they are referring to the ontological hierarchy. However, we foresee that for future use cases we might add more and shorter labels for those classes to accommodate developers and users. In OMIABIS the MIABIS attributes are given as "alternative name" for the class in question.

### Performance of OMIABIS regarding the competency questions

Table 1 shows the DL queries we executed using the DL query tab of Protégé and their results. The test ontology based on OMIABIS and populated with example individuals performed flawlessly in answering the competency questions as specified in the Methods section.

## Discussion

### OMIABIS in relation to pre-existing efforts in biobank ontology

Ontologies have been identified as a key technology to overcome the lack of semantic integration of biobank-related data [21]. [3] demonstrates how pre-existing ontologies, namely the Ontology of Biomedical Investigation (OBI) [22] and BioTop [23] can be efficiently used to represent data regarding samples and sample curation in a semantically rich way. The methodology used, and the criteria applied, by [3] overlap with our approach to ontology development. In our research we focused on administrative data regarding biobanks, sample collections, and studies producing sample collections, whereas [3] focuses on individual specimens or samples. We plan to use a similar approach and integrate their work in subsequent research that will address the issue of properties of individual samples [24]. Developed an ontology-based architecture to integrate data from heterogeneous biobanks by unifying metadata. Since the outcome of their development is not open source, we contacted the developers and aim to cooperate with them on the OMIABIS project.

Another ontology that represents biobanks/biorepositories is the eagle-i resource ontology (ERO)^r^, which was created for the eagle-i project. The aim of the eagle-i project is to "create a searchable inventory of unique, rare or otherwise hard-to-find biomedical resources … to foster sharing and linking of resources in the larger scientific community". ERO is used to integrate data about biomedical resources and make the search functionality more flexible [25]. However, due to its use case ERO is relatively sparse with respect to axiomatic representation of its classes. Our goal was to provide a semantically rich ontology that allows extensive reasoning, so re-use of ERO classes was not an option. In addition, we found ambiguities and lack of clarity in its representation of biobanks, specifically the fact that it defines biobank organization instead of biobank.. We have since begun collaborating with the ERO developers on the branches of ERO related to biobanks and their management.

### Performance of OMIABIS regarding the competency questions

The fact that all true positives were retrieved and none of the false positives was, hints to the fact that the ontology performs well. Based on our timing results when running the queries, we suspect that the axiomatic definition of "biobank" (given in Results section) is computationally "expensive". Relatively simple queries that used this class ran slower that complex queries that did not refer to it.

We are aware that the number of individuals in the competency test ontology is small. Both (1) the initial use cases from BBMRI and (2) the usage of OMBIABIS in i2b2, which we present below, include federated search in multiple databases. This raises the question of how the ontology will be used to query across large data sets. Our scenarios focus on researchers retrieving data about possible sources of specimens (BBMRI) or specific specimens (i2b2) to do research. This task is part of a study's planning phase. It is not related to patient-related activities or the performance of lab work. Thus, we believe, it is reasonable to provide the researcher with the benefit of a federated search at the cost of speed. The query results could be sent to the researcher once they are available. There does not seem to be the need for immediate recall. Nonetheless, we do want to keep reasoning time to a minimum once we start running queries on large data sets. We therefore plan to implement or develop methods to ensure timely recall.

### Ontological challenges regarding the MIABIS attribute "biobank type"

Taking into consideration the immediately biobank-related attributes in MIABIS, we found one attribute to be challenging from the perspective of ontology development: *biobank type*. Among the values for this attribute in MIABIS are for example *Pathology, Cytology, Gynecology* etc*.* There are strong indications from MIABIS users that this list is not exhaustive. The rationale behind this attribute and its current values is to allow the person submitting data about a biobank to easily select something that seems plausible to her. However, the downside of this approach is a certain difficulty for end users to find relevant biobanks and studies for her research. The possible values for biobank type in MIABIS are under elaboration and will be updated as time progresses. A particular specimen collection, by virtue of the type of specimens stored, might be of interest to both pathologists and virologists, or gynecologists and cytologists, and so on. In order to provide useful ontological representation of these classes we need users to specify which characteristics of a biobank make it useful for which specialty of medicine or which research domain.

### Using OMIABIS to annotate data in i2b2

In addition to putting OMIABIS to use within the BBMRI framework, we plan to use it for biobank data management at University of Arkansas for Medical Sciences (UAMS) and the Arkansas Children’s Hospital Research Institute (ACHRI). UAMS has a Tissue Procurement Facility and several, relatively smaller individual research labs (i.e. the Myeloma Institute, the "Spit for the Cure" Project). In addition, ACHRI has several independent labs similarly managing specimens, including the Center for Birth Defects Research, Section of Developmental-Behavioral and Rehabilitative Pediatrics (autism research), and the Women's Mental Health Program. Both UAMS and ACHRI would like to share their collected specimens and annotated data for research purposes while keeping the operations of each lab independent. Recently, UAMS created an Enterprise Data Warehouse (EDW) to facilitate access to and integration of clinical, basic-science, and other data for research and quality reporting. Retrieving de-identified data from the EDW is done using Informatics for Integrating Biology and the Bedside (i2b2) [26,27], an open-source software application. i2b2 was designed primarily for cohort identification, allowing users to perform queries to determine the existence of a set of patients meeting certain inclusion or exclusion criteria. Researchers have requested adding the ability to search for specimens to the data warehouse.

To ensure semantic integration of data from multiple biobanks with research relevant patient data, i2b2 requires an ontology to which the data will be mapped in i2b2's Ontology Cell. Because the management, the operations, and the data collected in the biobanks are heterogeneous, manual mapping of the data into a single i2b2 instance is a challenge. Instead, a federated architecture where queries are distributed to individual nodes and the results merged is the more promising approach. This approach requires a common ontology like OMIABIS.

Currently the biobanks at UAMS use caTissue [28], an open-source biospecimen management tool. caTissue is developed under the cancer Biomedical Informatics Grid (caGRID) initiative of the National Cancer Institute (NCI). It facilitates the process of locating and analyzing tissue specimens by cancer researchers based on clinical, tissue, and genomic characteristics. caTissue Annotation forms store clinical and other related data about specimens. Also called Dynamic Extensions, this component allows the creation of new forms that contain fields a site wishes to collect about each specimen.

**Figure 2:**
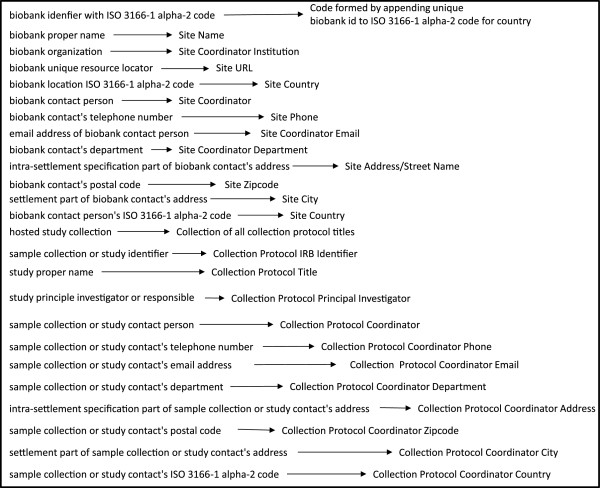
Mapping between OMIABIS classes and caTissue data elements.

Despite using a single software application, integration of data is not guaranteed in this approach because each biobank creates its own specimen annotation forms with different data elements. To ensure and optimize semantic integration, we will incorporate an ontology into caTissue’s annotation forms for all UAMS/ACHRI biobanks and the biobank administration data model. Then, the data in separate caTissue instances for the biobanks can be easily incorporated into the EDW i2b2 instance, and queried with common semantics. The researchers running the EDW have identified OMIABIS as the ontology it will use for biobank data. Figure 2 shows the mapping of OMIABIS terms to caTissue data elements previously used by UAMS' EDW.

McCusker et al. [29] have studied an option that would convert NCIt curated Unified Modelling Language (UML) annotations to OWL using semCDI. semCDI query formulation uses a view of caBIG semantic concepts, metadata, and data as an ontology [30]. The result was that OWL annotation properties are used to represent metadata on OWL constructs and are not considered for reasoning purposes. So, McCusker et al. have indeed created their own UML-to-OWL transformation that does not model attributes as datatype properties and does not model NCIt annotations of UML classes using subsumption. This methodology limits the expressivity and limits reasoning ability. In addition, this approach did not consider multiple biobanks.

To fulfill all requirements of biobank data integration within the UAMS/ACHRI framework, in the future OMIABIS representations will need to be integrated with ontologies representing individual specimens and donors.

Our next step is to cooperate with other biobank projects and biobank ontologies to extend OMIABIS and to work towards a domain ontology for biobanking as a whole. OMIABIS will be curated and maintained as an open-source artifact using subversion on an ongoing basis, with periodic releases of new versions.

## Conclusions

In conclusion, we created OMIABIS, an ontology of biobank administration. We found that basing its development on pre-existing resources to meet the BBMRI use cases resulted in a biobanking ontology that is re-useable in environments other than BBMRI.. With respect to answering the competency questions, our queries against an OMIABIS-based ontology, populated with a small set of hypothetical test cases, retrieved only true positives and did not miss any true positives. In addition, the mapping to a pre-existing data structure in the open-source caTissue application used for biospecimen collections in a medical center in Little Rock, AR demonstrated the adequacy of the coverage of OMIABIS.

## Endnotes

^a^http://purl.obolibrary.org/obo/obi.owl

^b^http://translationalmedicineontology.googlecode.com/svn/trunk/ontology/tmo.owl

^c^http://apps.who.int/classifications/icd10/browse/2010/en

^d^The Public Population Project in Genomics (P^3^G): http://www.p3g.org.

^e^The International Society for Biological and Environmental Repositories (ISBER): http://www.isber.org.

^f^Basic Formal Ontology (BFO): http://ifomis.org/1.1

^g^Principles of the OBO Foundry: http://obofoundry.org/crit.shtml

^h^The Proper Name Ontology (PNO): http://purl.obolibrary.org/obo/iao/pno.owl

^i^The Information Artifact Ontology (IAO): http://purl.obolibrary.org/obo/iao.owl

^j^The Ontology of Biomedical Investigation (OBI): http://purl.obofoundry.org/obo/obi.owl

^k^The Ontology of Medically Related Social Entities (OMRSE): http://purl.obolibrary.org/obo/omrse.owl

^l^The Protégé Ontology Editor and Knowledge Acquisition System: http://protege.stanford.edu

^m^HermiT Reasoner: http://www.hermit-reasoner.com

^n^classes printed bold, *object properties* in italics and OPERATORS all caps. Definitions of classes referred to here can be found in Table 1

^o^Note that this class description is based on object properties and classes from BFO, IAO and OBI.

^p^http://purl.obolibrary.org/obo/OBI_0000245

^q^The 'transfers' object property is represented in Document Acts Ontology (d-acts): http://purl.obolibrary.org/obo/iao/d-acts.owl

^r^The eagle-i Resource Ontology (ERO): http://purl.obolibrary.org/obo/ero.owl

## Competing interests

The authors declare that they have no competing interest.

## Authors' contributions

MB is the creator of the OWL file, provided the ontology-related background of the paper and edited the paper in its entirety. In addition, he provided the competency test. MB & WRH did the main ontological analysis of the domain and authored the OWL implementation. MNF, ME, RMM, LN, SK, MH, UT, WRH, JEL contributed to the ontology development and reviewed the ontology. RAH provided the method calculating the reasoning times and ran the measurements. NVK and UT provided the mapping to i2b2 and its integration into i2b2. All authors reviewed and commented on the paper until there was agreement. All authors read and approved the final manuscript.

## Supplementary Material

Additional file 1 OMIABIS Competency Test. Click here for file
